# Healthy behaviors awareness and the knowledge-practice gap among pregnant university students: an explanatory sequential study on quality of life and support services

**DOI:** 10.3389/fpubh.2026.1863913

**Published:** 2026-09-03

**Authors:** Amnah A. Aldohan, Ahmed G. Ismail, Hany M. Hagar, Hanan M. Diab, Usama M. Ibrahem

**Affiliations:** 1 College of Humanities and Social Sciences, Princess Nourah Bint Abdulrahman University, Riyadh, Saudi Arabia; 2 Department of Sports Sciences and Physical Activity, College of Education, UOH, Hail, Saudi Arabia; 3 Department of Educational Technology, University of Hail-KSA, Hail, Saudi Arabia; 4 Department of Educational Technology, Suez Canal University, Ismailia, Egypt

**Keywords:** health behaviors, healthy habits, pregnant students, quality of life (QoL), reproductive healthcare

## Abstract

**Background:**

Pregnant female university students face overlapping health, academic, and psychosocial demands, which can impact their quality of life and their ability to apply health knowledge in their daily lives. This study aimed to examine awareness of health behaviors, the gap between knowledge and practice, perceived quality of life, and institutional support needs among pregnant female university students.

**Methodology:**

A sequential interpretive research design with mixed methodologies was employed. Quantitative data were collected from 500 pregnant female students across nine Saudi universities using validated health behavior and quality of life measures. Qualitative interviews were conducted after the quantitative data collection to interpret patterns emerging from the statistical results.

**Results:**

The students reported a very high level of awareness of health behaviors during pregnancy. However, this awareness was not consistently reflected in practice. Quality of life varied, with general health and time management receiving more positive ratings than emotional and family-related areas. Qualitative findings indicated that academic pressure, limited dedicated support, social expectations, and inconsistent access to pregnancy-related services all contributed to the gap between knowledge and practice.

**Conclusion:**

Awareness alone is insufficient to ensure healthy practices among pregnant university students. Universities should develop integrated support models that combine health education, flexible academic policies, counseling, and referral mechanisms to maternal health services.

## 1 Introduction

Reproductive healthcare is a crucial component of a pregnant woman’s health and quality of life. Its importance is amplified for pregnant university students due to the overlap and increasing demands of pregnancy with academic and family obligations during this period. Pregnant students not only face the physical changes of pregnancy but also the challenges of academic pressure, attendance requirements, assessment demands, and family and social obligations as wives, which can lead to anxiety, stress, and difficulty adjusting to both roles simultaneously (1–3).

Research indicates that students can continue their education during pregnancy more easily when they have access to clear and transparent academic accommodations, comprehensive psychological support, health counseling, and flexible attendance and assessment options (4–6). Universities that provide individualized study plans, confidential support, and referral services for appropriate care help pregnant students maintain their academic progress and overcome the challenges of academic difficulties (7, 8). Studies show that prenatal services, nutritional counseling, psychological support, health education, financial assistance, and a supportive university environment can alleviate anxiety, improve psychosocial adjustment, and positively impact quality of life during pregnancy (6). Conversely, the absence of these services or limited access to them makes female students more vulnerable to stress, isolation, and difficulty balancing the demands of pregnancy and studies (9, 10).

These challenges are particularly significant because health awareness often does not translate into practical action. Pregnant students frequently know what to do healthily but fail to adhere to it due to academic pressures, university and family restrictions, or weak community support (9). Therefore, the gap between knowledge and practice is a key interpretive approach to understanding healthy pregnancy behaviors among female students, especially in university environments with varying levels of support and facilitation (7). The perception of QoL during pregnancy is not merely a health dimension but a multidimensional experience encompassing physical health, psychological stability, academic satisfaction, family relationships, and feelings of security and support (8, 11). The World Health Organization clarifies that QoL reflects an individual’s perception of their place in life within the context of their culture, values, goals, and expectations—a highly appropriate definition for understanding the experience of pregnant female students within the university environment (3, 12, 13).

Therefore, the current study addresses a research gap: the limited number of studies that have comprehensively examined the relationship between health awareness, the gap between knowledge and practice, quality of life, and institutional support among pregnant female students in the university environment, particularly within the Saudi context. The available evidence also points to the need for a holistic approach that considers physical, psychological, social, and educational dimensions simultaneously, rather than simply describing health awareness in isolation from actual application conditions.

The current study aims to examine the level of awareness among pregnant female students regarding health behaviors, identify the most important support services available to them within the university, measure their perceived quality of life during pregnancy in the university environment, and explore the relationship between these variables in light of social, demographic, and institutional support factors. It also seeks to provide deeper insights into the knowledge-practices gap, thereby helping universities develop support services that are tailored to the needs of pregnant female students.

## 2 The questions

(1) What is the level of awareness and practice of healthy behaviors during pregnancy among pregnant university students?
(2) To what extent are pregnant university students aware of a gap between awareness of health behaviors and their actual application?
(3) What is the perceived quality of life among pregnant university students across its main dimensions?
(4) What is the relationship between awareness of prenatal health behaviors and the different dimensions of quality of life among pregnant university students?
(5) How are awareness and practice of health behaviors related to quality of life, psychological adjustment, and academic and social support?

## 3 Framework

Pregnant university students occupy a unique position at the intersection of reproductive health requirements and academic responsibilities. The theoretical framework integrates three interrelated areas: maternal health awareness and behaviors, the QoL during pregnancy, and the structural and contextual determinants that shape health practices within the learning environment of higher education institutions. The theoretical framework was developed considering the research problem and questions to explain why awareness, if present, does not translate into behaviors and the importance of institutional support provided by universities to foster behaviors and a sense of well-being.

### 3.1 Health behaviors: the knowledge-practice gap

The knowledge, awareness, and practice gap in maternal health reflects a recurring pattern where awareness based on knowledge does not translate into recommended behaviors and, consequently, consistent adherence to them. Previous research (11) on pregnancy-related health behaviors has shown that women may know what is recommended, but they still face practical, social, or emotional challenges that limit its application and conversion into behavioral habits (14). This gap is particularly important for pregnant students, whose daily routines are impacted by academic workload, pregnancy-related needs, financial pressures, time constraints, and limited access to dedicated support (15).

The health beliefs model offers a valuable perspective for understanding this pattern, focusing on perceptions of risk or illness, perceived risk, perceived benefits, perceived challenges, self-efficacy, and motivation (16). According to this model, pregnant students are more likely to adopt healthy behaviors when they believe these behaviors are beneficial, easy to implement, and supported within their learning environments. However, academic demands, social stigma, or limited support within the university setting may hinder the acquisition of the necessary knowledge for sustainable practices.

### 3.2 Quality of life during pregnancy

QoL is defined as an individual’s perception of their position and roles in life considering their culture, societal values, personal goals, expectations, and interests. During pregnancy, this concept is multidimensional, encompassing the pregnant student’s physical health, emotional stability, family relationships, academic achievement, and perceived social support (17). For pregnant university students, QoL is not solely defined by health status but also reflects their ability to maintain academic achievement, manage stress and anxiety during pregnancy, and feel accepted within the university environment (11, 14).

Research findings indicate that social support provided to pregnant students is a strong predictor of subjective well-being during pregnancy and that university responses influence the experience of pregnancy in higher education (8, 18). Universities that offer academic flexibility, counseling services, health referrals, and comprehensive services are more likely to contribute to the well-being of pregnant students and mothers. Emotional and family dimensions often appear to be more sensitive to pregnancy-related stressors than physical ones, making them particularly relevant to this study (19).

### 3.3 Influencing socio-demographic factors

Socio-demographic factors may influence both the practice of healthy behaviors and the perceived quality of life of pregnant students. Age, academic level, and educational progression all influence coping ability, self-efficacy, and health literacy, which in turn affect how pregnant students respond to challenges (20). Older or more academically advanced students may have better access to coping strategies and greater familiarity with educational institutions, while younger or first-year students may face greater challenges, uncertainty, and reliance on external support (20).

These factors should be understood not only as background information but also as potential influences on the relationship between awareness and practice. This study helps explain why some students are more successful at applying knowledge practically than others and why perceived QoL varies among subgroups. This is particularly important when explaining differences in academic adjustment, emotional stability, and support needs.

### 3.4 University support as a contextual factor

Universities play a significant role in shaping the health and educational paths of pregnant students (11, 21). Recent policy documents and evidence emphasize the need to support pregnant students through inclusive and safe learning environments with flexible academic arrangements and access to health services (16). This support includes academic accommodations, psychological counseling, financial assistance, and referral channels to appropriate care.

In practice, institutional support from universities can reduce stress, improve academic achievement, and promote adherence to healthy behaviors by removing obstacles. The availability of such support is particularly important in contexts where pregnancy may be accompanied by social stigma or reduced social participation (16, 19). In the Saudi context, the alignment between university practices, cultural norms, and maternal well-being represents an important area of research that requires further investigation.

### 3.5 Conceptual integration

**Figure 1:**
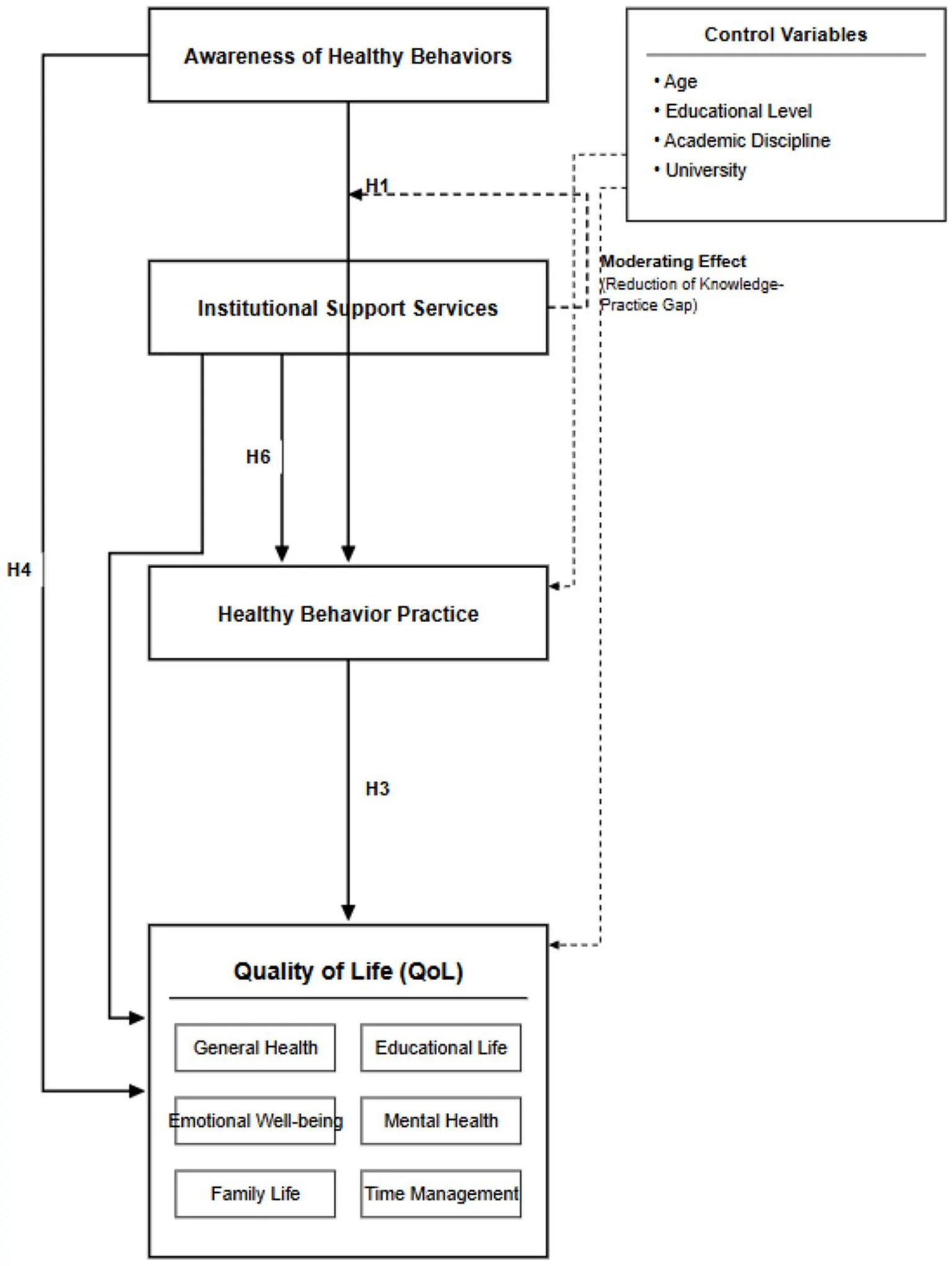
Comprehensive SEM framework of awarness, support service, healthy practice, and QoL.

The framework assumes that healthy behavior during pregnancy is shaped by the interaction of multiple factors. Figure 1 illustrates the integrated conceptual framework that guides this study, outlining the relationships between awareness of healthy behavior, the gap between knowledge and practice, social and demographic variables, institutional support, and QoL.

Figure 1 demonstrates that the understanding of healthy behaviors among pregnant students is anticipated to affect their practices, thereby enhancing their quality of life. The paradigm suggests a direct relationship between health awareness and quality of life, allowing additional study into whether awareness alone can improve outcomes. Moreover, institutional support services are anticipated to promote healthy behavioral patterns and enhance quality of life by alleviating practical obstacles that contribute to the disparity between knowledge and practice. Demographic and educational attributes, such as age, educational attainment, academic discipline, and university association, are regarded as contextual factors that may affect these correlations.

The conceptual framework, as illustrated in Figure 1, shows that practicing healthy behaviors is the central mechanism linking awareness of healthy behaviors to quality of life. Institutional support services are assumed to enhance this pathway by providing academic, health, and psychosocial resources that facilitate the adoption of recommended behaviors during pregnancy. The framework also acknowledges the potential influence of demographic and educational characteristics as contextual variables that may affect the strength of these links.

**Figure 2:**
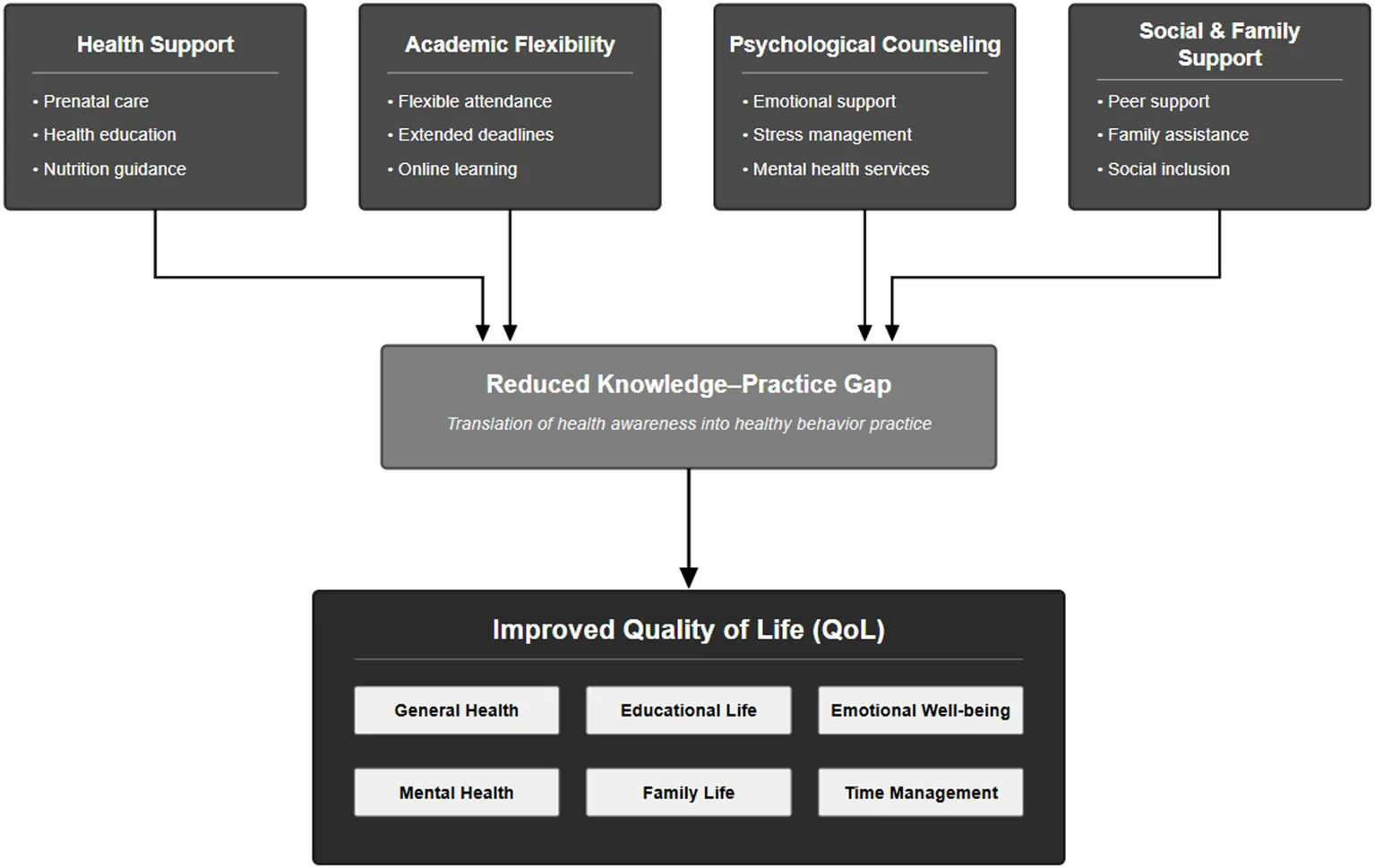
Integrated university support model for reducing the knowledge- practice gap and improving QoL among pregnant university students.

Based on the proposed conceptual framework and the evidence identified in the literature, an integrated university support model was developed to illustrate how institutional services can facilitate the translation of health awareness into healthy practices and ultimately improve the quality of life of pregnant university students, as shown in Figure 2.

As illustrated in Figure 2, university support is viewed as a multidimensional system encompassing health support, academic flexibility, psychological counseling, and social and family support. These services collectively contribute to reducing the barriers that prevent pregnant students from applying their health knowledge in their professional lives. By bridging the gap between knowledge and application, this model is expected to improve multiple aspects of QoL, including overall health, educational experience, emotional well-being, mental health, family life, and time management.

## 4 Methodology

This study followed the sequential interpretive mixed methods design described by Creswell and Plano-Clark (22), in which the quantitative results guided the subsequent qualitative phase.

The study relied on the descriptive analytical approach to monitor and extrapolate the published intellectual production related to the most important services provided to pregnant female students in universities, their most important needs during this period, awareness of healthy behavior during pregnancy and its relationship to quality of life, and making regional and international verbal comparisons by relying on documentary research based on collecting and analyzing data and sources related to published literature.

As for the applied aspect, the mixed approach was relied upon, using the Explanatory Sequential Design strategy, where the research team collected quantitative data and analyzed it in the first phase of data collection, followed by collecting qualitative data and analyzing it in the second phase. This is to obtain deeper data about the phenomenon, which relies on questionnaires to obtain information related to the subject of the study and analyze it according to quantitative and qualitative statistical methods.

### 4.1 Participant

Table 1 showed the participants, which consisted of 500 pregnant female university students from 9 universities, aged between 18 and 23 years, with an average age of 22.4 years and a standard deviation of 0.96 years.

**Table 1:** Participant characteristics.

Variable	Levels	Frequency	Percentage
Level	Fourth	220	44%
	Third	140	28%
	Second	110	22%
	First	30	6%
Major	Engineering	85	22.4%
	Humanities	195	51.3%
	Medical	100	26.3%
The university	Hail	60	12%
	King Saud	65	13%
	King Khalid	40	8%
	Taybah	50	10%
	Qassim	60	12%
	Tabuk	60	12%
	King Abdulaziz	50	10%
	Al-Jouf	30	6%
	Princess Nourah bint Abdulrahman	95	19%

### 4.2 Tools

#### 4.2.1 First: prenatal maternal health behavior scale among pregnant university students

To validate the scale, confirmatory factor analysis was performed on a sample of 500 female students using the maximum likelihood method. The results showed an acceptable to excellent fit for the model; the chi-square value was χ^2^ = 3,670 with df = 90 degrees of freedom, and the statistical significance was less than 0.001. The following fit indices were also recorded: RMSEA = 0.048, SRMR = 0.086, CFI = 0.996, TLI = 0.996, NNFI = 0.996, and IFI = 0.996. These values indicate a very acceptable to excellent fit between the model and the data, supporting the construct validity of the scale.

The results also showed that all item saturations on the overall factor were statistically significant, ranging from 0.565 to 1.048, reflecting a high degree of structural consistency in the scale. The reliability coefficient using Cronbach’s alpha was 0.945, while the McDonald omega coefficient was 0.951, indicating a high degree of internal consistency in the scale (Table 2).

**Table 2:** Validity and reliability indicators.

Valu	Χ2	df	p	RMSEA	SRMR	CFI	TLI	NNFI	IFI	Cronbach’s Alpha	McDonald’s Omega
Indicator	3,670	90	<0.001	0.048	0.086	0.996	0.996	0.996	0.996	0.945	0.951

The structural picture of the scale becomes clearer through the item loads on the overall factor, as shown in Table 3.

**Table 3:** Scale item saturations on the overall factor.

Item	Saturation	Standard error	Z-value	Significance
1	0.999	0.022	214.5	<0.001
2	0.970	0.011	162.9	<0.001
3	0.9477	0.006	67.6	<0.001
4	0.806	0.015	62.7	<0.001
5	0.965	0.005	25.2	<0.001
6	0.976	0.014	285.0	<0.001
7	0.794	0.045	58.8	<0.001
8	0.945	0.014	153.2	<0.001
9	0.978	0.022	159.4	<0.001
10	0.880	0.011	152.3	<0.001
11	0.999	0.006	87.6	<0.001
12	0.945	0.013	97.9	<0.001
13	0.765	0.045	162.9	<0.001
14	0.956	0.007	289.6	<0.001
15	1.028	0.003	157.8	<0.001
16	0.880	0.006	73.4	<0.001
17	0.937	0.023	67.5	<0.001
18	1.048	0.006	53.1	<0.001
19	0.970	0.014	151.5	<0.001
20	0.880	0.003	153.2	<0.001
21	0.565	0.007	151.0	<0.001

The results in Tables 3, 4 confirm that the scale possesses good psychometric properties. The conformity indices supported construct validity, while the reliability coefficients demonstrated high internal consistency. All items contributed significantly to explaining the overall factor, thus supporting the scale’s suitability for use.

**Table 4:** Quality of life scale for pregnant university students.

Value	No. of items	No. of dimensions	Χ^2^	df	*p*	RMSEA	SRMR	CFI	TLI	NNFI	IFI	Cronbach’s Alpha	McDonald’s Omega
Indicator	23	1	573.4	252	<0.001	0.058	0.072	0.963	0.961	0.961	0.963	0.924	0.931

#### 4.2.2 Second: the quality-of-life scale for pregnant university students scale

This scale measures a pregnant student’s sense of physical comfort, psychological satisfaction, and ability to continue her university life during pregnancy and includes the physical, psychological, academic, and environmental dimensions related to her university experience.

To assess the quality of the scale (as in Table 3), two confirmatory factor analyses (CFAs) were performed using the maximum likelihood estimation method on the entire study sample (*n* = 380). The fit quality indices for the Quality-of-Life Scale for Pregnant University Students scale showed acceptable to excellent fit: χ^2^(df) = 114, *p* < 0.001; RMSEA = 0.058, indicating good fit to the model (23); SRMR = 0.072; and all incremental fit indices were greater than 0.95 (CFI = 0.963, TLI = 0.961, NNFI = 0.961, and IFI = 0.963). Although the chi-squared statistic was statistically significant—an expected result in large samples (n > 200)—the χ^2^/df ratio remained below the recommended threshold of 3.0 (24), supporting the model’s efficiency. Internal consistency was confirmed by Cronbach’s alpha (*α* = 0.924) and McDonald’s omega (*ω* = 0.931), both of which significantly exceed the 0.70 threshold recommended by Nunnally and Bernstein (25).

#### 4.2.3 Third: academic and social support scale for pregnant female university students

This scale measures the level of support a pregnant student receives from family, friends, colleagues, faculty members, and academic advisors and the extent of social and academic support available to her during pregnancy.

To assess the quality of the scale (as in Table 5), two confirmatory factor analyses (CFAs) were performed using the maximum likelihood estimation method on the entire study sample (*n* = 380). The fit quality indices for the Academic and Social Support Scale for Pregnant University Students scale showed acceptable to excellent fit: χ^2^(df) = 11, *p* < 0.001; RMSEA = 0.056, indicating good fit to the model (23); SRMR = [0.068]; and all incremental fit indices were greater than 0.95 (CFI = 0.971; TLI = 0.968; NNFI = 0.968; IFI = 0.971). Although the chi-squared statistic was statistically significant—an expected result in large samples (n > 200)—the χ^2^/df ratio remained below the recommended threshold of 3.0 (24), supporting the model’s efficiency. Internal consistency was confirmed by Cronbach’s alpha (*α* = 0.911) and McDonald’s omega (*ω* = 0.918), both of which significantly exceed the 0.70 threshold recommended by Nunnally and Bernstein (25).

**Table 5:** Academic and social support scale for pregnant university students.

Valu	No. of items	No. of dimensions	Χ^2^	df	*p*	RMSEA	SRMR	CFI	TLI	NNFI	IFI	Cronbach’s Alpha	McDonald’s Omega
Indicator	12	1	134.7	65	<0.001	0.056	0.068	0.971	0.968	0.968	0.971	0.911	0.918

#### 4.2.4 Fourth: psychological adjustment scale for pregnant female university students

This scale measures the degree of emotional balance and the ability to cope with stress, anxiety, sadness, and loneliness and the extent of general psychological adaptation to pregnancy and university studies.

To assess the quality of the scale (as in Table 6), two confirmatory factor analyses (CFAs) were performed using the maximum likelihood estimation method on the entire study sample (n = 380). The fit quality indices for the Psychological Adjustment Scale for Pregnant Female University Students scale showed acceptable to excellent fit: χ^2^(df) = 10, *p* < 0.001; RMSEA = 0.056, indicating good fit to the model (23); SRMR = 0.074; and all incremental fit indices were greater than 0.95 (CFI = 0.961; TLI = 0.958; NNFI = 0.958; IFI = 0.961). Although the chi-squared statistic was statistically significant—an expected result in large samples (n > 200)—the χ^2^/df ratio remained below the recommended threshold of 3.0 (24), supporting the model’s efficiency. Internal consistency was confirmed by Cronbach’s alpha (*α* = 0.935) and McDonald’s omega (*ω* = 0.941), both of which significantly exceed the 0.70 threshold recommended by Nunnally and Bernstein (25).

**Table 6:** Psychological adjustment scale for pregnant university students.

Valu	No. of items	No. of dimensions	Χ^2^	df	*p*	RMSEA	SRMR	CFI	TLI	NNFI	IFI	Cronbach’s Alpha	McDonald’s Omega
Indicator	20	1	416.3	189	<0.001	0.056	0.074	0.961	0.958	0.958	0.961	0.935	0.941

#### 4.2.5 Fifth: prenatal maternal health behaviors scale for pregnant female university students

This scale measures actual health behaviors related to pregnancy in college students, including nutrition, physical activity, medical follow-up, prevention, stress management, and adherence to health care guidelines.

To assess the quality of the scale (as in Table 7), two confirmatory factor analyses (CFAs) were performed using the maximum likelihood estimation method on the entire study sample (*n* = 380). The fit quality indices for the Prenatal Maternal Health Behavior Scale among pregnant university students showed acceptable to excellent fit: χ^2^(df) = 2.21, *p* < 0.001; RMSEA = 0.057, indicating good fit to the model (23); SRMR = 0.071; and all incremental fit indices were greater than 0.95 (CFI = 0.968, TLI = 0.965, NNFI = 0.965, and IFI = 0.968). Although the chi-squared statistic was statistically significant—an expected result in large samples (n > 200)—the χ^2^/df ratio remained below the recommended threshold of 3.0 (24), supporting the model’s efficiency. Internal consistency was confirmed by Cronbach’s alpha (α = 0.948) and McDonald’s omega (ω = 0.951), both of which significantly exceed the 0.70 threshold recommended by Nunnally and Bernstein (25).

**Table 7:** Prenatal maternal health behavior scale among pregnant university students.

Valu	No. of items	No. of dimensions	Χ^2^	df	*p*	RMSEA	SRMR	CFI	TLI	NNFI	IFI	Cronbach’s Alpha	McDonald’s Omega
Indicator	15	1	229.6	104	<0.001	0.057	0.071	0.968	0.965	0.965	0.968	0.948	0.951

## 5 The results

To answer the 1st question, “What is the level of awareness and practice of healthy behaviors during pregnancy among pregnant university students?” a one-sample t-test was used to determine the level of awareness of healthy behaviors during pregnancy, as shown in Table 8.

**Table 8:** One-sample t-test to determine the level of awarness of pregnant students about healthy habits.

*n*	Mean	Standard deviation	T value	Degrees of freedom	Significance	Effect size
500	68.6	9.43	57.5	499	0.000	3.21 High

The results showed that the students had a very high level of awareness of healthy behaviors, with a mean of 68.6 and a standard deviation of 9.43, reflecting a relatively similar level of awareness among the sample. The test results also showed a statistically significant difference between the actual and hypothetical means (t = 57.5, p < 0.001), with a very high effect size (d = 3.21), indicating that this high level is not only statistically significant but also has considerable practical importance. This result suggests that the students have a good understanding of health practices related to pregnancy, which may reflect the effectiveness of health education resources and community awareness campaigns focused on maternal health. This finding is consistent with a number of previous studies that confirmed the high level of health knowledge among pregnant women, while a gap continues to exist between knowledge and the actual application of health behaviors, which supports the need for university interventions aimed at transforming health awareness into sustainable daily practices (15, 17, 26).

*To answer the 2nd question: “To what extent are pregnant university students aware of a gap between awareness of health behaviors and their actual application?”* The weighted average and standard deviation of the sub-dimensions of the questionnaire on the services related to the quality of life provided to each of the pregnant female students in Saudi universities were used. The results were as in Table 9.

**Table 9:** The degree of achievement of the services provided with the QoL provided to pregnant female students.

Services	Weighted mean	Standard deviation
Emotional and psychological support services during pregnancy.	2.95	0.93
Academic accommodations during pregnancy,	2.0.82	0.83
Health care services.	2.95	0.88
Financial assistance related to pregnancy needs.	3.01	1.18
Social integration and campus environment.	2.78	1.01

Table 9 reveals that pregnant female university students are aware of a gap between their knowledge of healthy behaviors during pregnancy and their ability to practice these behaviors within the university environment. The average levels of service dimensions were generally at a moderate level, indicating that the problem is not solely related to a lack of health awareness, but also to insufficient supportive conditions that would help them translate this awareness into regular daily practice. Financial support related to pregnancy needs ranked first, followed by psychological and emotional support and healthcare, while academic facilities, social integration, and the campus environment ranked lower. This reflects that the most pronounced aspects of the gap are related to the student’s university and social environment.

The results in Table 10 illustrate the nature of this gap. In the psychological and emotional dimension, relatively high indicators of stress related to academic workload and feelings of isolation emerged, along with a clear lack of access to specialized psychological counseling services for pregnant students. This suggests that while students may be aware of the importance of psychological well-being and stress management during pregnancy, they often lack a structured institutional channel to help them cope with anxiety, stress, and the fear of conflict between pregnancy and academic demands. This finding aligns with the findings of Ojong et al. (6), Hoag et al. (10), and Duffy et al. (9), which indicated that the absence of psychological and social support makes pregnant students more vulnerable to stress, isolation, and difficulty balancing pregnancy and studies.

**Table 10:** The most important needs for services provided to pregnant women.

No.	Statements	Strongly agree	Agree	Neutral	Disagree	Strongly disagree	Mean	Standard deviation
Emotional and psychological support services during pregnancy								
1	Stress related to academic workload	69 13.8%	94 18.8%	140 28%	39 7.8%	158 31.6%	3.25	1.42
2	Anxiety about balancing study and pregnancy/childcare responsibilities	51 10.2%	11 2.2%	108 21.6%	238 47.6%	92 18.4%	2.38	1.12
3	Depression or feelings of isolation	19 3.8%	210 42%	67 13.4%	72 14.4%	132 26.4%	3.18	1.32
4	Counseling or mental health services specifically designed for pregnant students.	51 10.2%	24 4.8%	20 4%	320 64%	85 17%	2.27	1.12
Academic accommodations during pregnancy								
5	Flexible deadlines for assignments/exams	44 8.8%	100 20%	33 6.6%	114 22.8%	209 41.8%	3.69	1.41
6	Flexible scheduling options (online or hybrid classrooms-asynchronous learning).	275 55.2%	109 21.8%	64 12.8%	—	51 10.2%	1.88	1.26
7	Extended leave policies without academic penalties	—	40 8%	120 24%	122 24.4%	218 43.6%	4.04	1
8	Reasonable accommodations to help pregnant students make up work missed due to medical absences.	200 40%	23 4.6%	202 40.4%	24 4.8%	51 10.2%	2.41	1.32
9	Academic counseling specifically designed for pregnant students.	23 4.6%	191 38.2%	143 28.6%	77 15.4%	66 13.2%	2.94	1.12
Health care services								
10	Prenatal care services	147 29.4%	135 27%	167 33.4%	—	51 10.2%	2.35	1.20
11	Appropriate sexual education and contraceptives	128 25.6%	108 21.6%	109 21.8%	8 1.6%	147 29.4%	2.88	1.56
12	Nutrition counseling	91 18.2%	134 26.8%	175 35%	32 6.4%	68 13.6%	2.70	1.23
13	Family planning counseling	54 10.8%	116 23.2%	141 28.2%	19 3.8%	170 34%	3.27	1.41
14	On-campus screening for pregnancy-related complications	41 8.2%	231 46.2%	124 24.8%	53 10.6%	51 10.2%	2.68	1.10
15	Providing regular checkups to monitor fetal growth and maternal health	52 10.6%	11 2.2%	101 20.2%	143 28.6%	192 38.4%	3.82	1.26
Financial assistance is related to pregnancy needs								
16	Scholarships/aid for pregnant students	107 21.4%	74 14.8%	76 15.2%	136 27.2%	107 21.4%	3.12	1.46
17	Emergency funds for medical expenses	112 22.4%	106 21.2%	142 28.4%	—	140 28%	2.90	1.49
Social inclusion and campus environment								
18	Faculty support for pregnant students.	145 29%	139 27.8%	151 30.2%	14 2.8%	51 10.2%	2.37	1.22
19	Resist stigma or discrimination from peers or staff due to pregnancy.	53 10.6%	82 16.4%	158 31.6%	52 10.4%	155 31%	3.35	1.35
20	Provide programs or initiatives aimed at promoting inclusion.	130 26%	88 17.6%	204 40.8%	—	78 15.6%	2.62	1.30

Regarding academic accommodations, the results showed that the most pressing needs were flexible assignment and exam scheduling, extended leave policies without academic penalties, and compensation for missed classes due to absences. The findings indicate that the gap between awareness and practice widens when students are aware of the desired healthy behaviors but find themselves constrained by attendance requirements, exam pressure, and time constraints. This finding aligns with those of Ara et al. (4), Mendes et al. (5), and Saruchera and Chidarikire (7), who emphasized that the continued education of pregnant students requires flexible university policies, individualized study plans, and clear procedures that accommodate pregnancy without hindering academic progress.

In the area of healthcare, the results revealed a clear need to enhance regular follow-up services, nutritional counseling, and pregnancy screenings on campus or through established referral channels. This indicates that awareness of healthy behaviors is insufficient without readily accessible services, especially given busy academic schedules and the challenges of commuting or scheduling medical appointments. This finding supports the findings of Battulga et al. (15) and Boutib et al. (11), which demonstrated that health knowledge may not translate into action when pregnant women face practical, social, or institutional barriers that limit adherence to recommended practices.

The results also showed that social integration and the campus environment represent one of the weakest supportive aspects for pregnant students, indicating persistent challenges related to stigma, inadequate inclusion programs, and limited organized social support. This finding explains a significant aspect of the knowledge-practice gap; a student may be aware of what she needs to do health-wise but hesitate to seek help or disclose her needs due to social embarrassment or fear of judgment. This aligns with the findings of Vhumbunu (8) and Nguyen-Louie et al. (18), whose studies confirmed that social and institutional support is crucial for improving the quality of life and sense of security among pregnant female students.

Therefore, it can be confirmed that pregnant female university students are highly aware of a gap between health knowledge and its practical application. This gap stems not so much from a lack of knowledge as it is linked to the interplay of academic pressures, inadequate specialized psychological services, limited academic facilities, insufficient healthcare within the university, and weak social integration. This finding underscores the core of the study’s theoretical framework: knowledge only translates into sustainable practice when a supportive university environment is available that enhances self-efficacy, reduces barriers, and provides clear pathways for health, psychological, and academic support. Bridging the knowledge-practice gap requires an integrated institutional model that combines health education, academic flexibility, psychological counseling, healthcare, and financial and social support. This model contributes to improving the quality of life for pregnant female students and enhancing their ability to balance the demands of pregnancy with university life.

*To answer the 3rd question: “What is the perceived quality of life among pregnant university students across its main dimensions?”* a single-sample t-test was used to determine the level of feeling of pregnant students about aspects of quality of life during pregnancy, and the results were as shown in Table 11.

**Table 11:** One-sample t-test to determine the level of pregnant students’ feelings about aspects of quality of life during pregnancy.

Dimension	*n*	Mean	Standard deviation	T Value	Degrees of freedom	Sig.	Effect size
Quality of general health	500	42.5	5.55	70.68	499	0.000	3.16 High
Quality of education and study	500	28.2	8.17	8.85	499	0.000	0.396 Moderate
Quality of emotions	500	27.9	7.76	8.38	499	0.000	0.375 Moderate
Quality of mental health	500	31.5	7.25	20.06	499	0.000	0.897 High
Quality of time occupation and management	500	30.5	7.66	16	499	0.000	0.716 Moderate
Quality of family life	500	34.1	5.31	38.50	499	0.000	1.72 High
Total quality of life score	500	195	33.5	29.9	499	0.000	1.34 High

Table 11 shows that pregnant university students generally enjoy a high level of quality of life, with an overall mean of (195) and a high statistical significance (t = 29.90, *p* < 0.001) and a large effect size (d = 1.34). This indicates that the quality of life among the sample was significantly higher than the hypothetical mean. However, analysis of the sub-dimensions reveals that quality of life is not homogeneous but varies across health, psychological, academic, and social aspects.

General health came in first place, with a mean of (42.5) and a very high effect size (d = 3.16). This indicates that the majority of students feel physically healthy during pregnancy. This result can be explained by the high level of health awareness demonstrated by the results of the first question, in addition to the students’ interest in monitoring their pregnancy and adhering to basic health practices. This aligns with the findings of Oechsle et al. (26) and Battulga et al. (15), and Nare and Casper (17), who confirmed that increased health awareness contributes to improved self-perception of health during pregnancy.

The results also showed that the quality of mental health was among the high dimensions, with a mean of 31.5 and a large effect size (d = 0.897). This indicates that most students were able to maintain a reasonable level of psychological well-being despite the changes associated with pregnancy. This can be explained by the presence of various sources of family and social support, in addition to the ability of many students to adapt to the demands of this stage. This aligns with the findings of studies by Nguyen-Louie et al. (18) and Vhumbunu (8), which demonstrated that social and institutional support is one of the most important factors influencing the psychological well-being of pregnant students.

Conversely, the quality of family life was also high, with a mean of 34.1 and a large effect size (d = 1.72). This reflects the fact that the family environment is an important source of stability and support during pregnancy. This result is consistent with the literature that confirms that family support is one of the most important factors in helping students adapt to pregnancy and improve their quality of life, especially in societies characterized by strong family ties.

The quality of study and education was found to be at an average level (28.2), as was the quality of time management (30.5) and the quality of emotional well-being (27.9), indicating that these aspects continue to pose a challenge for pregnant students. The results show that pregnancy does not significantly affect students’ perception of their overall health, but it does increase the difficulty of balancing academic demands with health and family responsibilities. It also affects emotional well-being and time management more than it affects physical well-being. This aligns with the findings of Ara et al. (4), Mendes et al. (5), Alyousef et al. (21), and Saruchera and Chidarikire (7), which emphasized that flexible academic systems and university support are crucial factors in improving the educational experience for pregnant students. It also supports the findings of Duffy et al. (9) and Hoag et al. (10), which demonstrated that academic and social pressures are among the most significant factors affecting emotional stability during pregnancy.

The results indicate that pregnant female university students enjoy a good quality of life, although this level varies across the measured dimensions. Health aspects appear more stable compared to academic and emotional aspects. This finding supports the study’s theoretical framework, which views quality of life as a multidimensional concept influenced by the interaction of physical health, mental health, the academic environment, and social and institutional support. It also underscores that improving the quality of life for pregnant female students requires more than just providing healthcare; it necessitates developing more flexible university policies, enhancing psychological counseling services, and improving academic and social support programs to enable students to achieve a better balance between the demands of pregnancy and university life.

*To answer the 4th question, “What is the relationship between awareness of prenatal health behaviors and the different dimensions of quality of life among pregnant university students?,” Pearson’s correlation coefficient was used to analyze the correlation between the level of health awareness and the various dimensions of quality of life, as shown in* Table 12.

**Table 12:** Pearson correlation matrix for the relationships between pregnant students’ sense of quality of life and awareness of health behaviors.

		1	2	3	4	5	6	7
Awareness of health behaviors	1	—						
Quality of public health	2	0.854***	—					
Quality of family life	3	0.348***	0.372***	—				
Quality of education and study	4	−0.160	−0.176	0.595***	—			
Quality of emotions	5	0.025	0.030	0.815***	0.914***	—		
Quality of mental health	6	0.080*	0.062	0.757***	0.843**	0.932***	—	
Quality of time occupation & management	7	0.080*	0.069	0.662***	0.734***	0.761***	0.593***	—
Overall quality of life score	8	0.199***	0.218***	0.878***	0.874***	0.967***	0.906***	0.832***

* Significant at 0.05 significance level, ** Significant at 0.01 significance level, *** Significant at 0.001 significance level.

The results revealed a heterogeneous pattern of correlations between health awareness and the dimensions of quality of life. Health awareness was strongly positively correlated with overall health quality (r = 0.854, *p* < 0.001), the strongest correlation coefficient in the matrix, indicating that higher levels of health awareness are associated with improved perceptions of health during pregnancy. This finding aligns with the study’s theoretical framework, which posits that health awareness is the starting point for improved health and well-being and is consistent with the findings of Oechsle et al. (26), Battulga et al. (15), and Nare and Casper (17), who confirmed that health knowledge is associated with improved subjective health indicators among pregnant women.

Conversely, a moderate positive correlation emerged between health awareness and family quality of life (r = 0.348, *p* < 0.001), and a weak positive correlation with the overall quality of life score (r = 0.199, *p* < 0.001). This suggests that health awareness contributes to improved quality of life, but its impact remains limited when considering quality of life as a multidimensional concept influenced by health, academic, psychological, and social factors simultaneously.

The results also revealed very weak or practically non-significant correlations between health awareness and academic quality, emotional well-being, mental health, and time management, although some of these correlations were statistically significant. This indicates that possessing health knowledge alone is insufficient to improve these aspects, as they are influenced by organizational, psychological, and social factors that extend beyond individual knowledge. This interpretation is consistent with recent literature which has shown that academic pressures, lack of institutional flexibility, and poor university support services may limit the ability of pregnant students to leverage their health knowledge to enhance their educational and emotional experiences (4, 5, 7, 9, 10).

On the other hand, the correlation matrix revealed strong positive relationships between most dimensions of quality of life. Emotional quality of life was strongly correlated with both mental health (r = 0.932) and academic quality (r = 0.914). All dimensions were also highly correlated with the overall quality of life score, particularly emotional (r = 0.967), mental health (r = 0.906), and family life (r = 0.878). These results indicate that the quality of life of pregnant female students is a cohesive construct in which health, psychological, family, and academic dimensions interact in an integrated manner. This supports the theoretical framework adopted by the study, which views quality of life as a product of the interaction between health, psychological, family, and university environment factors, rather than the result of a single factor.

The results demonstrate that awareness of healthy behaviors is an important condition for improving quality of life, but it is not sufficient on its own. Translating this awareness into actual improvement in various dimensions of quality of life requires a supportive university environment that provides academic flexibility, health services, psychological counseling, and social support, thereby reducing the gap between health knowledge and practical application, which is consistent with the conceptual model proposed in the study.

*To answer the 5th question: “How are awareness and practice of health behaviors related to quality of life, psychological adjustment, and academic and social support?*

The results of the multivariate analysis of variance (MAVA) in Table 13 show that the effects of demographic and educational characteristics were not equal on the study variables. Academic level was found to have a statistically significant effect on the study variables (*F* = 9.94, *p* < 0.001), as was age (*F* = 7.73, p < 0.001). In contrast, neither the level nor the specialization of education or university showed a statistically significant effect. This suggests that the differences among the students are not primarily due to differences in university, level, or specialization, but rather are more closely related to factors such as maturity, experience, and academic advancement.

**Table 13:** Results of the multiple analysis of variance for the effect of demographic variables on the social study variables.

Source of variance	Hotelling’s t-test	*F*-value	Degree of freedom 1	Degree of freedom 2	Significance
Education Sector	0.008	0.91	4	894	0.458
Academic Level	0.009	9.94	4	894	0.000
University	0.003	0.144	1	894	0.999
Age	0.035	7.73	2	448	0.000

This result implies that older students or those with higher academic standing may be better equipped to understand the demands of pregnancy, manage their time effectively, and cope with academic and health pressures. This can be explained by the fact that older age and higher academic standing provide students with greater experience navigating the university environment, a better ability to seek help, and the capacity to make more informed health decisions. Younger students or those in the early grades may be more prone to confusion, lack of experience, and dependence on irregular external support.

The partial correlation results in Table 14 support this interpretation. After isolating the effects of age and academic level, the correlation between health awareness and overall health quality remained very strong and statistically significant (r = 0.851, *p* < 0.001). This indicates that the relationship between health awareness and overall well-being is stable and not solely dependent on age or academic level. Students with higher health awareness are often more likely to perceive their health status positively, even after adjusting for demographic differences.

**Table 14:** Analysis of the partial correlation matrix after isolating the effect of demographic variables.

		1	2	3	4	5	6	7
Awareness of health behaviors	1	—						
Quality of public health	2	0.851***	—					
Quality of family life	3	0.339***	0.378***	—				
Quality of education and study	4	−0.164	−0.167	0.595***	—			
Quality of emotions	5	0.017	0.037	0.815***	0.912***	—		
Quality of mental health	6	0.79*	0.074*	0.757***	0.838***	0.930***	—	
Quality of time occupation & management	7	0.057	0.059	0.662***	0.733***	0.756***	0.582***	—
Overall quality of life score	8	0.189***	0.223***	0.873***	0.874***	0.967***	0.904***	0.826***

* Significant at 0.05 significance level, ** Significant at 0.01 significance level, *** Significant at 0.001 significance level.

The relationship between health awareness and overall QoL score also remained statistically significant, albeit relatively weak (r = 0.189, *p* < 0.001). This confirms that health awareness contributes to improved QoL, but it is not sufficient on its own to explain it. The QoL of pregnant students is also influenced by family support, academic flexibility, healthcare, social integration, and stress management skills.

The results showed that some relationships between dimensions of QoL remained strong after isolating the effects of age and academic level. The strong relationship persisted between emotional quality and mental health, between academic quality and emotions, and between family life quality and the overall QoL score. This suggests that QoL among pregnant students is a coherent construct; emotional or academic distress can impact mental health, and family support can improve the overall sense of QoL.

These findings are consistent with the study’s theoretical framework, which posits that demographic and educational characteristics are not merely descriptive variables but contextual factors that influence a student’s ability to translate health awareness into practical action. They also align with the findings of Battulga et al. (15), Boutib et al. (11), Lee, et al. (27), and Kisanga et al. (28) which indicated that health knowledge may not translate into consistent behavior when students face academic, social, or institutional pressures. Furthermore, they are consistent with the research of Etini & Glory (29), Higgins, et al., (30), Nguyen-Louie et al. (18) and Vhumbunu (8), who confirmed that the QoL of pregnant students is affected by social and institutional support, and not by health factors alone.

Therefore, it can be argued that age and academic level are significant factors in explaining the differences among pregnant university students in health awareness, quality of life, and coping with pregnancy. The university or the education sector as a whole did not show a substantial impact, indicating that the need for support is not specific to any one institution but rather a general need requiring comprehensive university policies. The findings suggest the necessity of targeted support programs for younger students and those in their early academic years, including health counseling, academic guidance, psychological support, and follow-up services, so that coping ability is not solely dependent on personal maturity or academic experience.

### 5.1 Limitations

Although the findings of this research represent a valuable contribution to the field, they must be evaluated within the limitations of the study. The results were limited to 500 female students from nine Saudi universities; therefore, changing the sample or its location could lead to different results, potentially limiting the generalizability of the findings to other educational and cultural contexts. Furthermore, the research focused on currently pregnant students and did not include graduate students who experienced pregnancy during their studies. Additionally, faculty and staff may have their own perspectives on the services provided to pregnant students.

## 6 Conclusion

Pregnant students showed a very high level of awareness of healthy habits during pregnancy; the arithmetic mean of awareness scores was 63.7 with a standard deviation of 8.88, and the one-sample t-test showed a high t-value of 57.5 with a high statistical significance (*p* = 0.000), and the effect size value was 2.95, indicating a high practical significance. Pregnant students showed high personal awareness and responsibility for their health and their fetuses. The study showed that pregnant students know about healthy habits but often lack practical application. The results also showed that pregnant students feel differently about different aspects of quality of life during pregnancy. Therefore, the results recommended strengthening counseling and community programs to bridge the gap between knowledge and practice.

The results also confirmed the positive impact of effective health education programs on students’ awareness levels. Universities also support access to reliable information and encourage healthy behaviors. The results also indicated that cultural and social factors work to enhance awareness levels and promote healthy habits for a healthy birth. Factors such as age, education level, marital status, and socioeconomic background also affect awareness and practice levels.

For the outcomes of quality of life in the university environment for pregnant students, general health quality was the highest perceived quality by pregnant students, with a mean of general health quality of 42.4 with a standard deviation of 5.59. The high t-value (60.73) and large effect size (3.115) reflected the priority of health factors for pregnant students. Education and study quality ranked second with a medium level of perception, with a mean of education quality of 28.3 with a standard deviation of 8.25; the t-value was 7.86, but the effect size was low (0.403). The perception of emotional quality was low among pregnant students, with a mean of 28 and a standard deviation of 7.85. The t-value of emotional quality was 7.34 with a lower effect size (0.377), indicating negative emotional impact. Also, the perception of mental health quality was high, with a mean of 31.6 and a standard deviation of 7.30; The t-value was 17.52. The large effect size (0.899) indicated good psychological health among pregnant students despite psychological challenges. Also, the perception of quality time management was high, with a mean of 30.6 and a standard deviation of 7.70; the t-value was 14.11. The effect size for time management was (0.724), reflecting effective time management despite challenges. Meanwhile, family life quality ranked lowest in perception among pregnant students, with a mean of 34.2 and a standard deviation of 5.38. The t-value for family life quality was highly statistically significant at 33.18, but the effect size (0.392) was relatively small.

### 6.1 Recommendation

Universities should redesign health education as a continuous, on-demand service, rather than simply informational activities that meet quality standards. Based on Bandura’s theory of social cognitive learning, it is recommended to establish sustainable guidance programs that include healthy nutrition, psychological support, and prenatal health education. The research team believes these programs can be practically implemented by creating a dedicated unit within the Deanship of Student Affairs to support pregnant students. This unit would offer scheduled weekly counseling sessions and on-demand counseling services via flexible, remote digital platforms that fit within study schedules. A university application could also be developed to provide personalized content and track healthy behaviors.

Enhancing the impact of these programs requires comprehensive support. In light of Bronfenbrenner’s ecosystem theory, universities should adopt an institutional approach that includes providing specialized psychological counseling services, establishing peer support networks, and appointing academic advisors trained in maternal issues on campus. The implementation of these efforts can be delegated to specific entities within the university, such as the Deanship of Student Affairs (for overall coordination), the University Medical Center (for health services), the Psychological Counseling Units, and the Deanship of Admissions and Registration (for academic flexibility).

To ensure measurability and evaluability, these efforts should be linked to clear performance indicators, such as increased adherence to periodic medical checkups, improved health behavior indicators (before/after intervention), reduced stress levels, and increased service utilization rates. Longitudinal designs can be adopted to track these indicators across different academic years through university quality units, thus enhancing the accuracy of assessment and the sustainability of improvement.

Healthy behaviors should be encouraged through a supportive university environment, rather than solely focusing on individual responsibility. This can be achieved by providing healthy food options on campus, physical activity programs specifically designed for pregnant women, and developing financial support mechanisms, including grants for pregnant students, partial tuition waivers, and on-campus childcare services. Regarding the implementation timeline, a phased plan is recommended, including short-term measures (0–6 months) such as launching basic counseling services; medium-term measures (6–18 months) to build partnerships with healthcare providers; and long-term measures (over 18 months) to integrate these services into formal university policies.

Collaboration between universities and community healthcare providers is crucial for enhancing the effectiveness of interventions. In light of social capital theory, it is recommended to establish formal agreements with hospitals and clinics to provide screening and follow-up services on campus, thereby reducing access barriers and promoting continuity of care. Cultural specificity and local context should be considered when designing these interventions, including respecting privacy, ensuring family participation, and aligning with the policies of the Ministries of Education and Health.

To improve the responsiveness of programs to beneficiaries’ needs, it is recommended to involve pregnant students in the design and evaluation of services through the activation of volunteer clubs, focus groups, and periodic surveys. Based on the above, the study recommends a shift from traditional outreach models to a comprehensive, evidence-based, socially and culturally appropriate, and measurable institutional support model. The pregnant university student is not an exceptional case, but rather an indicator of the university’s commitment to providing equitable, inclusive, and health-promoting learning environments.

### 6.2 Future directions

Future research directions stem from the study’s central finding: the persistent gap between health knowledge and practice among pregnant university students, coupled with a low quality of emotional life. This underscores the need to shift research from descriptive approaches to more precise, contextualized, and interpretive interventions.

Longitudinal studies are a methodological priority. It is recommended to adopt longitudinal cohort designs that track the same sample across pregnancy and postpartum, using standardized tools such as the WHOQOL Quality of Life Index, periodic questionnaires, and health records. This would allow for an analysis of the evolution of the knowledge-practice gap and an assessment of the cumulative impact of institutional support and academic facilities on health outcomes. Furthermore, social support should be repositioned as a key explanatory variable, given the weakness in the emotional dimension revealed by the study. This can be achieved by operationally analyzing its role (number and frequency of interactions, type of support: family/peer/academic) and evaluating its role as a mediating variable using mediation analysis models. This would enable an assessment of the effectiveness of interventions such as peer mentoring or family involvement in improving health behaviors. In a related context, digital technologies represent a promising field; however, their effectiveness requires rigorous empirical testing. This involves designing pilot studies that measure the impact of health applications on tracking nutrition, physical activity, and mental health, while distinguishing between raising awareness and effecting actual behavioral change, given the structural constraints identified in the study. Furthermore, the findings of no differences between universities regarding the impact of age and academic level raise questions about the effectiveness of institutional frameworks. This necessitates comparative studies linking university policies with student outcomes at the national level.

## Data Availability

The raw data supporting the conclusions of this article will be made available by the authors, without undue reservation.
